# Tumor Heterogeneity in Human Epidermal Growth Factor Receptor 2 (HER2)-Positive Advanced Gastric Cancer Assessed by CT Texture Analysis: Association with Survival after Trastuzumab Treatment

**DOI:** 10.1371/journal.pone.0161278

**Published:** 2016-08-12

**Authors:** Sung Hyun Yoon, Young Hoon Kim, Yoon Jin Lee, Jihoon Park, Jin Won Kim, Hye Seung Lee, Bohyoung Kim

**Affiliations:** 1 Department of Radiology, Seoul National University Bundang Hospital, Seoul National University College of Medicine, Institute of Radiation Medicine, Seoul National University Medical Research Center, Seongnam-si, Korea; 2 Division of Hematology and Medical Oncology, Department of Internal Medicine, Seoul National University Bundang Hospital, Seoul National University College of Medicine, Seongnam-si, Korea; 3 Department of Pathology, Seoul National University Bundang Hospital, Seongnam-si, Korea; 4 Division of Biomedical Engineering, Hankuk University of Foreign Studies, Yongin-si, Korea; Taipei Medical University, TAIWAN

## Abstract

**Background:**

Image texture analysis is a noninvasive technique for quantifying intratumoral heterogeneity, with derived texture features reported to be closely related to the treatment outcome of tumors. Gastric cancer is one of the most common tumors and the third leading cause of cancer-related deaths worldwide. Although trastuzumab is associated with a survival gain among patients with human epidermal growth factor receptor 2 (HER2)-positive advanced gastric cancer, optimal patient selection is challenging. The purpose of this study was to determine whether CT texture features of HER2-positive gastric cancer were related to the survival rate after trastuzumab treatment.

**Methods and Findings:**

Patients diagnosed with HER2-positive advanced gastric cancer from February 2007 to August 2014 were retrospectively selected. Using in-house built software, histogram features (kurtosis and skewness) and gray-level co-occurrence matrices (GLCM) features (angular second moment [ASM], contrast, entropy, variance, and correlation) were derived from the CT images of HER2-positive advanced gastric cancer in 26 patients. All the patients were followed up for more than 6 months, with no confirmed deaths. The patients were dichotomized into a good and poor survival group based on cutoff points of overall survival of 12 months. A receiver-operating characteristics (ROC) analysis was performed to test the ability of each texture parameter to identify the good survival group. Kaplan–Meier curves for patients above and below each threshold were constructed. Using a threshold of >265.8480 for contrast, >488.3150 for variance, and ≤0.1319×10^−3.^ for correlation, all of the area under the ROC curves showed fair accuracy (>0.7). Kaplan–Meier analysis showed statistically significant survival difference between two groups according to optimal cutoff values of contrast, variance, correlation and ASM. However, as this study had a small number of patients, a further study with a larger population will be needed to validate the results.

**Conclusions:**

Heterogeneous texture features on CT images were associated with better survival in patients with HER2-positive advanced gastric cancer who received trastuzumab-based treatment. Therefore, texture analysis shows potential to be a clinically useful imaging biomarker providing additional prognostic information for patient selection.

## Introduction

Intratumoral heterogeneity is a well-known feature of malignant tumors that can affect response rates and drug resistance to both cytotoxic and targeted chemotherapy [1, 2]. The use of image texture analysis as an noninvasive imaging biomarker for the identification of intratumoral heterogeneity has been introduced [1, 3]. Studies have shown that the results of image texture analysis were useful in the assessment and/or prediction of the tumor therapeutic response in various cancers, such as esophageal cancer, colorectal cancer, nonsmall cell lung cancer, and squamous cell carcinoma of the head and neck [4–9]. In primary esophageal cancer, low entropy and high uniformity values measured on contrast-enhanced CT images after chemoradiation therapy were associated with improved survival [4]. Several studies demonstrated an association between the response rate or prognosis after treatment and the texture features obtained from CT and MR images of patients with colorectal cancer [6, 7].

Gastric cancer is one of the most common tumors and the third leading cause of cancer-related deaths worldwide [10]. Early gastric cancer can be cured by surgical resection, but advanced cases have a poor prognosis, despite curative resection, followed by chemotherapy, including various cytotoxic agents [11]. In patients with stage IV gastric cancer, the median overall survival is less than 12 months, with a combination of chemotherapy consisting of various cytotoxic agents [12]. Recently, a randomized trial reported that the use of trastuzumab, a monoclonal antibody to human epidermal growth factor 2 (HER2) receptor to treat patients with HER2-positive gastric cancer conferred a significant survival [11]. However, not all the patients with HER2-positive gastric cancer showed a good response to trastuzumab [11]. This result implies that the optimal method for patient selection needs to be developed for targeted therapy in patients with HER2-positive gastric cancer.

To the best of our knowledge, no studies have investigated the use of texture analysis in gastric cancer, especially HER2-positive gastric cancer. Therefore, the purpose of this study was to determine whether CT texture features of HER2-positive gastric cancer are associated with the survival rate after trastuzumab treatment.

## Patients and Methods

### Patients

This study was approved by Institutional Review Board of Seoul National University Bundang Hospital, IRB No.: B-1509-316-101. This retrospective study utilized deidentified patient data collected from electronic medical records. Patients diagnosed with HER2-positive advanced gastric cancer at Seoul National University Bundang Hospital from February 2007 to August 2014 were included. Age, gender, Eastern Cooperative Oncology Group (ECOG) performance status, tumor location, pathological subtype, and documented HER2 status by immunohistochemistry (IHC) and fluorescence in situ hybridization (FISH) were collected by reviewing electronic medical records and evaluated. HER2-positive gastric cancer was defined as primary gastric HER2 IHC 3+ or IHC 2+/FISH-positive cancer (HER2/CEP17 ratio of ≥2) [11, 13]. Forty-three patients who had HER2-positive stage IV gastric cancer and received trastuzumab-based combination chemotherapy were identified. Among these patients, 17 were excluded from this study: 15 of the patients were excluded because they underwent a pretreatment CT examination at other hospitals, and the other two patients were excluded because primary gastric cancer could not be defined on their CT examinations. Thus, 26 patients with HER2-positive advanced gastric cancer who received trastuzumab-based combination chemotherapy were included. All these patients were followed up for more than 6 months, without any confirmed deaths.

### CT Examination

Contrast-enhanced CT examinations were performed using 16- (*n* = 4), 64- (*n* = 17), or 256- (*n* = 5) detector-row scanners (Mx 8000, Brilliance 64, or iCT256; Philips Medical Systems, Cleveland, OH). Each patient was asked to drink 1000 mL of tap water for gastric distension 10 min before the CT examination. Intravenous nonionic contrast material (2 mL/kg; iomeprol, Iomeron 350; Bracco, Milano, Italy) was administered via the antecubital vein, using a power injector (Stellant D, Medrad, Indianola, PA) at a rate of 3 mL/s. Bolus-tracking software (Brilliance; Philips Medical Systems) was used to trigger the scanning 60 s after the aortic enhancement reached a threshold of 150-HU. Helical scan data were acquired using 16×1.5, 64×0.625, or 128×0.625 mm collimation; a rotation speed of 0.5 s; a pitch of 1.25, 0.641, or 0.993; and a kvP of 120 kVp). Using an automatic tube current modulation technique (Dose-Right; Philips Medical Systems), effective mAs ranged from 69 to 379 mAs. Transverse and coronal section datasets were reconstructed with 4-mm thick sections at 3-mm increments.

### Texture Analysis

The archival contrast-enhanced CT studies were retrieved from a workstation of Picture Archiving and Communication System in Digital Imaging and Communications in Medicine file format. The texture analysis was performed using in-house built software. The polygonal region of interest (ROI) was manually drawn along the margin of the gastric cancer on the image slice displaying the tumor in its largest cross-sectional area, based on a consensus of two radiologists (Y.H.K., and S.H.Y., with 19 and 3 years of experience in abdominal oncological imaging, respectively) blinded to the clinical information (mean area of ROI, 1414 pixels; range of ROI, 174–3730 pixels). Preliminary image thresholding was performed to remove any pixels with attenuation values below -50 HU to exclude areas of air. The heterogeneity within the ROI was quantified using both histogram features and gray-level co-occurrence matrices (GLCM) features [14, 15]. The histogram features included kurtosis (magnitude of pixel distribution) and skewness (skewness of pixel distribution). The GLCM features included the angular second moment (ASM) (pixel repetition/orderliness representing the homogeneity of an image), contrast (local variation), entropy (randomness of the matrix), variance (difference from the mean), and correlation (brightness interdependence on neighboring pixels).

### Statistical Analysis

To determine the relationship between the CT texture features of the gastric cancer and overall survival, the patients were dichotomized into a good survival group (median overall survival longer than 12 months) versus a poor survival group (medial overall survival of less than 12 months). The cutoff points for overall survival of 12 months were estimated on the basis of median survival reported in previous studies [12, 13, 16]. A receiver-operating characteristics (ROC) analysis was performed to test the ability of each texture parameter to identify the good survival group. The point on the ROC curve furthest from the line of no-discrimination was considered the optimum threshold (maximum of the Youden index). Kaplan–Meier curves for patients above and below each threshold were constructed. Differences between the curves were evaluated by a log-rank test. The χ^2^ test, Fischer’s exact test, and Mann–Whitney *U* test were used to compare the clinicopathological parameters between the good and poor survival groups. A *p* value of <0.05 was considered significant. All the statistical analyses were performed by MedCalc 12.1.4.0 (MedCalc Software, Mariakerke, Belgium) and SPSS version 21 (SPSS, Chicago, IL).

## Results

### Patients

The study population consisted of 26 (24 men and 2 women; mean age ± standard deviation, 63 years ± 12; range, 42–80 years) patients. The median follow-up duration was 18.6 months (range, 0.2–50.8 months). The median overall survival was 18.6 months (95% confidence interval, 14.7–25.5 months). There were 18 patients assigned to the good survival group and 8 patients assigned to the poor survival group. Table 1 summarizes the patients’ characteristics. The age, sex, tumor location, and pathological type of tumor were not significantly different between the two groups. The ECOG performance status of the poor survival group was significantly lower than that of the good survival group. The median overall survival and median follow-up duration were significantly longer in the good survival group than the poor survival group.

**Table 1:** Patients’ characteristics.

	Total	Good survival	Poor survival	*P* value
	(*n* = 26)	(*n* = 18)	(*n* = 8)	
Age (range)	63 (42–80)	63.1 (42–80)	61.8 (46–74)	0.644
Sex				
Male	24 (92.3%)	16 (88.9%)	8 (100%)	1.000
Female	2 (7.7%)	2 (11.1%)	0 (0%)	
ECOG				
0	2 (7.7%)	1 (5.6%)	1 (12.5%)	
1	20 (76.9%)	17 (94.4%)	3 (37.5%)	
2	3 (11.5%)	0 (0%)	3 (37.5%)	
3	1 (3.8%)	0 (0%)	1 (12.5%)	0.026
Tumor location				
Gastroesophageal junction	3 (11.5%)	3 (16.7%)	0 (0%)	
Other stomach	23 (88.5%)	15 (83.3%)	8 (100%)	0.529
Pathology type				
Adenocarcinoma	25 (96.2%)	17 (94.4%)	8 (100%)	
Signet ring cell	1 (3.8%)	1 (5.6%)	0 (0%)	1.000
Other	0 (0%)			
Follow-up duration				
Median months (range)	18.6 (0.2–50.8)	24.3 (13.5–50.8)	5.4 (0.2–10.5)	<0.001
Overall survival				
Median months (95% CI)	18.6 (14.7–25.5)	24.3 (21.9–31.9)	5.4 (2.5–7.7))	<0.001

Note.–ECOG = Estern Cooperative Oncology Group, CI = confidence interval.

### Survival Analysis

**Fig 1:**
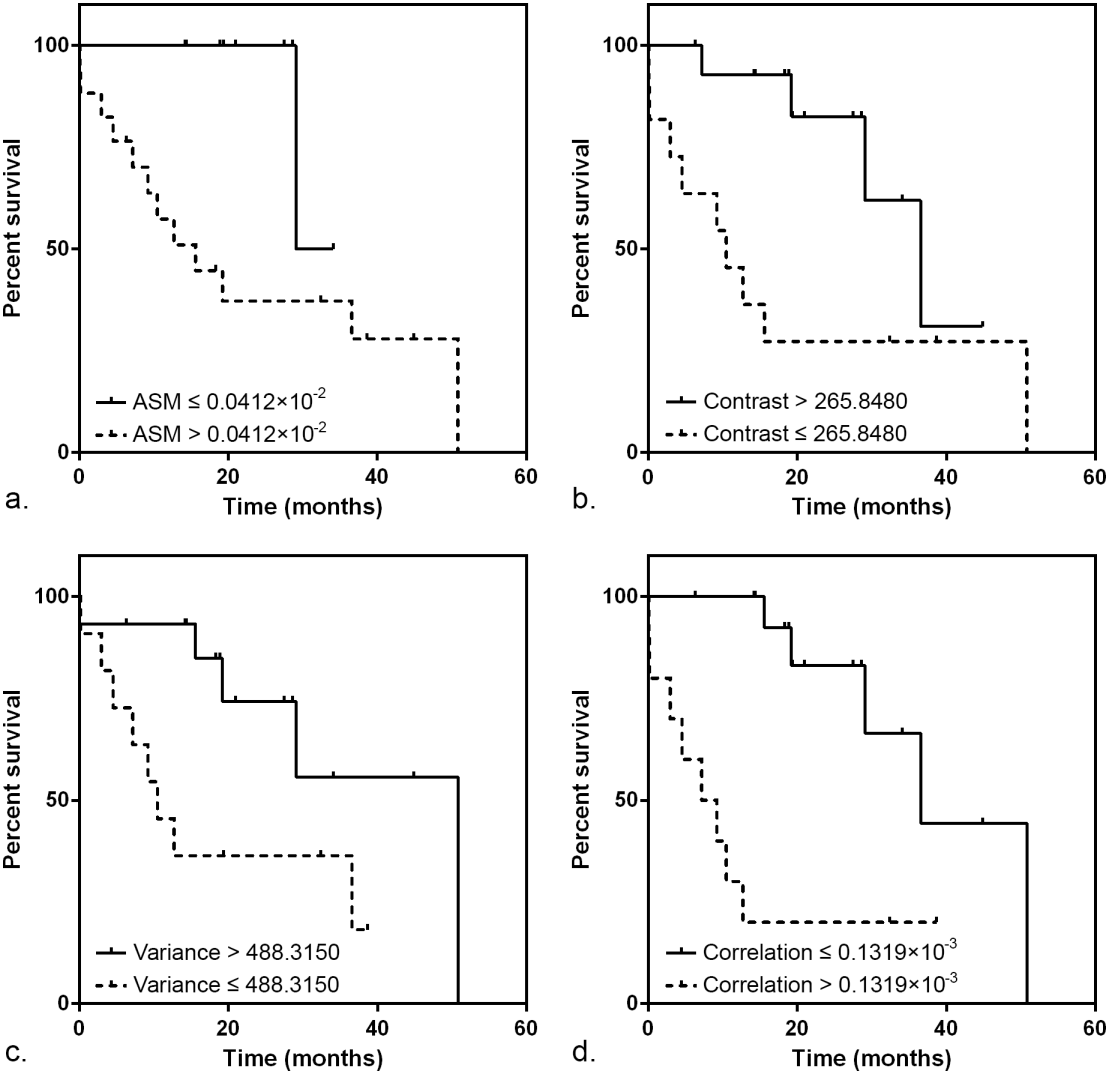
Kaplan–Meier curves according to texture features. Kaplan–Meier curves showed a significant difference in survival according to (a) ASM, (b) contrast, (c) variance, and (d) correlation, with log-rank *P* values of .027, .023, .037, and .001, respectively.

Details of measured texture features are provided as S1 Table. The results of the derived ROC threshold values for texture are summarized in Table 2. Using a threshold of >265.8480 for contrast, >488.3150 for variance, and ≤0.1319×10^-3.^for correlation, the area under the ROC curves (AUC) were all over 0.7, showing fair accuracy for the prediction of good survival, and all were statistically significant. Optimal thresholds of different texture features showed wide range of sensitivity and specificity, representing their different way to calculate textural characteristics. However, contrast, variance, and correlation showed similar results of sensitivity and specificity (Range: 72.22–83.33 and 72.22–87.50, respectively). Using the derived ROC threshold, Kaplan–Meier survival analysis was performed (Fig 1, Table 3). Texture features including the contrast, variance, and correlation that showed fair accuracy for the prediction of good survival in the ROC analysis also showed significant survival differences between the two groups divided by an optimal cutoff value. There were significant between-group differences in survival according to the ASM, when divided by an optimal cutoff value of ≤0.0412×10^−2.^.

**Table 2:** Results of the derived ROC threshold values for texture.

Texture variable	ROC threshold	Sensitivity (%)	Specificity (%)	AUC	95% CI	*P* value
ASM	≤0.0412×10^−2^	50.00	100.00	0.639	0.429–0.816	0.260
Contrast	>265.8480	72.22	87.50	0.771	0.565–0.911	0.004
Entropy	>7.7984	61.11	87.50	0.611	0.402–0.794	0.346
Variance	>488.3150	72.22	72.22	0.750	0.543–0.897	0.011
Correlation	≤0.1319×10^−3^	83.33	87.50	0.771	0.565–0.911	0.013
Kurtosis	≤0.6805	100.00	25.00	0.556	0.349–0.748	0.659
Skewness	≤-0.1092	55.56	75.00	0.597	0.388–0.783	0.437

**Table 3:** Results of the Kaplan–Meier survival analysis.

Texture variable	ROC threshold	Median survival in months (number of patients)		*P* value
		Above threshold	Below threshold	
ASM	≤ 0.0412×10^−2^	15.6 (17)	29.1 (9)	0.027
Contrast	> 265.8480	36.6 (15)	10.5 (11)	0.023
Entropy	>7.7984	29.1 (13)	36.6 (13)	0.691
Variance	> 488.3150	50.8 (15)	10.5 (11)	0.037
Correlation	≤ 0.1319×10^−3^	7.2 (10)	36.6 (16)	0.001
Kurtosis	≤0.6805	3.0 (3)	36.6 (23)	0.366
Skewness	≤-0.1092	19.2 (14)	29.1 (12)	0.505

## Discussion

The present study evaluated CT texture features in 26 patients with HER2-positive stage IV advanced gastric cancer who had received trastuzumab-based chemotherapy. In this study, tumor contrast greater than 265.8480, variance greater than 488.3150, correlation less than or equal to 0.1319×10^−3^, and an ASM less than or equal to 0.0412×10^−2^ were associated with good overall survival. In general, a higher contrast, higher variance, lower correlation, and lower ASM represent increased heterogeneity within a tumor [7, 14].

The ground-breaking international phase III Trastuzumab for Gastric Cancer (ToGA) trial, led to the approval of trastuzumab as first-line therapy, in combination with chemotherapy, for patients with HER2-positive advanced gastric cancer, and its use is now standard practice [11, 17]. Initially, the ToGA trial included only patients with HER2 IHC 3+ or HER2 FISH+ (HER2/CEP17 ratio of ≥2) advanced gastric cancer, but the post-hoc subgroup analysis of the trial showed that trastuzumab treatment was more beneficial to HER2 IHC 3+ or IHC 2+/FISH+ groups [11]. Subsequent studies focused on the histopathological HER2 status to select optimal patients for trastuzumab treatment and revealed that the HER IHC status, HER2/CEP17 ratio, and HER2 gene copy number were associated with good clinical outcomes in trastuzumab treatment [16, 18]. A previous study reported that a high incidence of intratumoral HER2 heterogeneity may result in an inaccurate assessment of the HER2 status and suboptimal patient selection [19, 20]. In other words, random sampling or biopsies to determine the histopathological HER2 status are not sufficient to represent the full extent of tumor. The present study included only patients with HER2 IHC 3+ or IHC 2+/FISH+ who had shown a good response to trastuzumab treatment. However, the survival of the patients still varied widely. This result implies that a method for optimal patient selection needs to be developed for targeted therapy in patients with HER2-positive gastric cancer. Assessment of tumor heterogeneity using texture analysis is an emerging imaging tool in clinical practice. Texture analysis can provide additional information about the tumor environment, such as cell proliferation, hypoxia, and angiogenesis, and the texture features from the analysis can serve as noninvasive imaging biomarkers that reflect intratumoral heterogeneity [3, 5, 8, 9]. Several studies proposed that increased tumor heterogeneity was associated with poor clinical outcomes. In a study of 54 patients with primary non-small lung cancer (NSCLC), uniformity measured on the unenhanced CT component of PET-CT was inversely correlated with poor survival [8]. A study of 21 patients with primary esophageal cancer undergoing PET-CT staging proposed that low uniformity and high entropy values were predictors of reduced survival [5]. In a study of 72 patients with locally advanced squamous cell carcinoma of the head and neck, increased entropy and skewness on contrast-enhanced CT images were associated with poor overall survival [9]. Potential biological correlates of radiological feature of heterogeneity are not clear. However, it has been hypothesized that the heterogeneity measured on CT might reflect the complex vascular microenvironment associated with tumor-induced angiogenesis [21, 22]. Recent studies of patients with NSCLC and colorectal cancer revealed a positive correlation between tumor heterogeneity at the medium and coarse texture scale on CT and hypoxia, supporting this hypothesis [21, 22].

In the current study, CT texture features reflecting increased tumor heterogeneity were associated with good overall survival. Therefore, it implied that HER2-positive advanced gastric cancer demonstrating heterogeneous texture features was associated with good overall survival. HER2 is a transmembrane tyrosine kinase receptor and member of the ErbB family. It can heterodimerize with other members of the ErbB family, which induce autophosphorylation of tyrosine residues in the cytoplasmic domain of the receptors and subsequently initiate a downstream signaling cascade, leading to cell-cycle progression, proliferation, survival, and ultimately tumorigenesis [23]. A previous study of the HER2 status in breast cancer suggested that HER2 overexpression was closely associated with the presence of necrosis and a high number of mitoses [24]. Another contrast-enhanced MR-based texture analysis study of breast cancer reported that HER2 overexpression groups show a significant increase in the entropy values that represent increased tumor heterogeneity [25]. In contrast to the findings of previous studies, which found that increased tumoral heterogeneity was associated with poor prognosis in lung, esophageal, and head and neck cancers [5, 8, 9], in the present study, tumors with increased heterogeneity, as measured by CT texture analysis, were associated with better overall survival. In patients with HER2-positive advanced gastric cancer, HER2 overexpression could strongly induce cell proliferation, with accompanying angiogenesis and necrosis. These tumoral features could explain the increased heterogeneity detected in the CT texture analysis. In the present study, the increased tumor heterogeneity measured by texture analysis represents not only true clonal heterogeneity, which is a well-known cause of therapeutic resistance, but also overall features of the tumoral microenvironment determined by HER2 overexpression, including cell proliferation, angiogenesis, and necrosis. This explains the association between the increased tumor heterogeneity detected in the CT texture analysis and the improved overall survival rate when treated with trastuzumab.

The present study contains several limitations. First, this study had a retrospective design, and the CT images were obtained by various CT machines. Thus, there could be a selection bias. Second, the study consisted of a small number of patients. Further validation with larger series is needed to improve confidence in the cutoff values of texture features. Multivariate analysis will be possible in the study with larger population and should be adjusted for potential clinical prognostic factor such as ECOG performance status. Third, survival analysis was done in the same population from which optimal cutoff values for survival analysis were derived. It could overestimate the prognostic value of the texture features. Lastly, we evaluated tumor heterogeneity in the largest cross-sectional area instead of whole-tumor analysis. Although whole tumor analysis may represent more diverse components of the of tumor heterogeneity [7], the measured entropy representing tumor heterogeneity was similar between both methods of measurement [26]. In our study, the measured texture features on the image slice displaying the tumor in its largest cross-sectional area could successfully show the patient’s prognosis.

In conclusion, heterogeneous texture features on CT images were associated with better survival in patients with HER2-positive advanced gastric cancer who received trastuzumab-based treatment. Therefore, texture analysis shows potential to be a clinically useful imaging biomarker providing additional prognostic information for patient selection.

## Supporting Information

S1 Table Details of measured texture features. (XLSX)
